# The effect of acupuncture needle combination on central pain processing-an fMRI study

**DOI:** 10.1186/1744-8069-10-23

**Published:** 2014-03-25

**Authors:** Albert Leung, Yi Zhao, Shivshil Shukla

**Affiliations:** 1 Department of Anesthesiology, The University of California, School of Medicine, 9300 Campus Point Drive, MC 7651, La Jolla, CA 92037, USA; 2 Department of Anesthesiology, Veterans Administration San Diego Healthcare System, 3350 La Jolla Village Drive, San Diego, CA 92161, USA; 3 The University of California, San Diego, 9500 Gilman Drive, La Jolla, CA 92037, USA

**Keywords:** TMM, Tendinomuscular meridian, Acupuncture, Analgesia, Analgesic mechanisms, fMRI, Electroacupuncture

## Abstract

**Background:**

Empirical acupuncture treatment paradigm for acute pain utilizing Tendinomuscular Meridians (TMM) calls for the stimulation of Ting Points (TPs) and Gathering point(GP). This study aims to compare the supraspinal neuronal mechanisms associated with both TPs and GP needling (EA3), and TPs needling alone (EA2) with fMRI.

**Results:**

A significant (P < 0.01) difference between pre-scan (heat Pain) HP, and post-EA HP VAS scores in both paradigms was noted (n = 11). The post-EA HP VAS score was significantly (P < 0.05) lower with EA3 comparing to EA2 Within-group random effect analysis indicated that EA3+HP>EA3 (condition EA3+HP subtracted by condition EA3) appeared to exert a significant degree of activity suppression in the affective supraspinal regions including the IPL, anterior cingulate cortex (ACC) and the insular cortex (IN). This level of suppression was not observed in the EA2+HP>EA2 (condition EA2+HP subtracted by condition EA2) within-group random effect analysis Between-group random effect analysis indicated that EA3 induced a significantly (P < 0.01, cluster size threshold 150) higher degree of deactivation than EA2 in several pain related supraspinal regions including the right prefrontal cortex, rostral anterior cingulate (rACC), medial cingulate cortex, left inferior frontal lobe and posterior cerebellum. The 2-factor ANOVA in those regions indicated both rACC and posterior cerebellum had a significant (P < 0.01) needle effect, and the right prefrontal area showed a significant (P < 0.01) HP effect. However, a significant interaction between the two factors was only found in the right prefrontal lobe. Granger causality analysis showed EA3 induced a much higher degree of inference among HP related supraspinal somatosensory, affective and modulatory components than EA2. Deactivation pattern at the medullary-pontine area casted a direct inference on the deactivation pattern of secondary somatosensory cortices which also affected the deactivation of the IN.

**Conclusions:**

While both EA2 and EA3 induced a significant degree of deactivation in the human brain regions related to pain processing, the addition of GP stimulation further exerts an inhibitory effect on the ascending spinoreticular pain pathway. Therefore, different needling position as mandated in different empirical acupuncture treatment paradigms may play a different role in modulating pain related neuronal functions.

## Introduction

With the increased utilization of acupuncture in pain management, a better understanding in the neuronal mechanisms underlying the observed analgesic benefit of acupuncture is required. As in most empirical acupuncture treatment paradigms, the tendinomuscular meridian (TMM) paradigm requires a set of acupuncture needle combination to treat acute pain in the extremities [1]. Recently, a series of studies was conducted to assess the neuronal mechanisms related to the needle combination in the TMM paradigm and the observed analgesic effect. The empirical TMM acupuncture paradigm for acute pain in the medial aspect of the lower extremities requires simultaneous needle stimulation of two classes of acupuncture points: the Ting Points (TPs) including LR1 (Big Mound) and SP1 (Yinbai), and the Gathering point (GP) called CV2 (Qugu Ren-2) [2]. Recently the analgesic function of these two classes of needling location was studied. For experimental thermal pain introduced at the medial aspect of lower extremities, electroacupuncture (EA) at TPs appeared to have analgesic effect at the ipsilateral limb in a dermatomally correlated distribution with a correlated deactivitional effect on the supraspinal pain processing network [3,4]. On the other hand, simultaneous stimulation of TPs and GP appeared to provide more sustainable analgesic effect in both lower extremities than TPs stimulation alone [5]. However, the specific supraspinal mechanisms related to the observed enhanced analgesic effect has not been previously studied. Given the anatomical difference in these two classes of acupuncture points, here we hypothesize that the added GP stimulation may provide additional pain inhibitory modulation via the afferent/ascending pain pathways [5]. This study aims to compare the supraspinal neuronal mechanisms associated with both TPs and GP needling (EA3), and TPs needling alone (EA2) with fMRI.

## Results

With the institutional Human Subject Review Committee approval, 11 healthy subjects (6 males, average age of 30.1 ± 5.2 years old) completed the study. This number of subjects is in line with the amount of subjects required in several previously published thermal pain related fMRI studies [6,7]. The average pre-scanning thresholds (°C ± SD) for cold, warm, cold pain and hot pain were 28.0 ± 1.7, 36.1 ± 2.1, 12.2 ± 10.2 and 48.5 ± 1.2 respectively. These baseline thermal thresholds of the subjects participating in the current study were within the normal range in reference to previously published studies [8,9]. The average heat pain (HP) visual analogue scale (VAS) scores (±SD) of the pre-scan HP, post-electroacupuncture (post-EA HP) for EA2 and EA3 paradigms were 41.0 ± 8.0, 15.1 ± 4.2 and 8.5 ± 3.9 respectively. Paired samples T-test (corrected) showed a significant (P < 0.01) difference between pre-scan HP, and post-EA HP VAS scores in both paradigms. The post-EA HP VAS score was significantly (P < 0.05) lower with EA3 comparing to EA2 . The result suggested both TMM treatment paradigms were effective in modulating HP. However, EA3 was more effective than EA2 in modulating HP perception. The deqi VAS scores were 55.2 ± 7.5 and 68.6 ± .6.4 for the EA2 and EA3 respectively.

### fMRI data analyses

**Figure 1:**
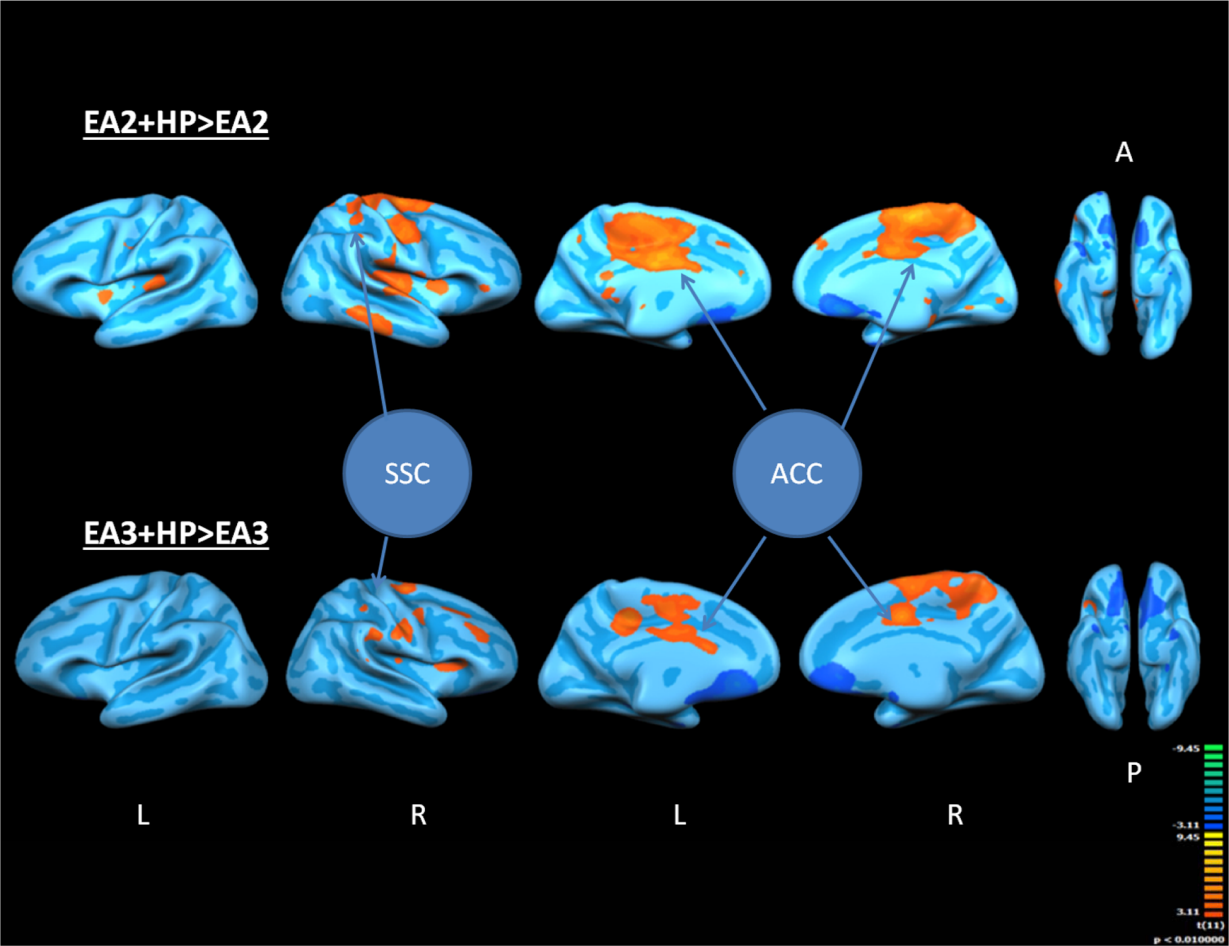
**Visual comparison of EA2+HP>EA2 with EA3+HP>EA3 paradigms.** EA2: Ting Points Stimulation; EA3 Gathering Point Stimulation; HP: Hot Pain; A: Anterior; P: Posterior; L: Left Hemisphere; R: Right Hemisphere; ACC: Anterior Cingulate cortex; SCC: Somatosensory Cortex.

**Figure 2:**
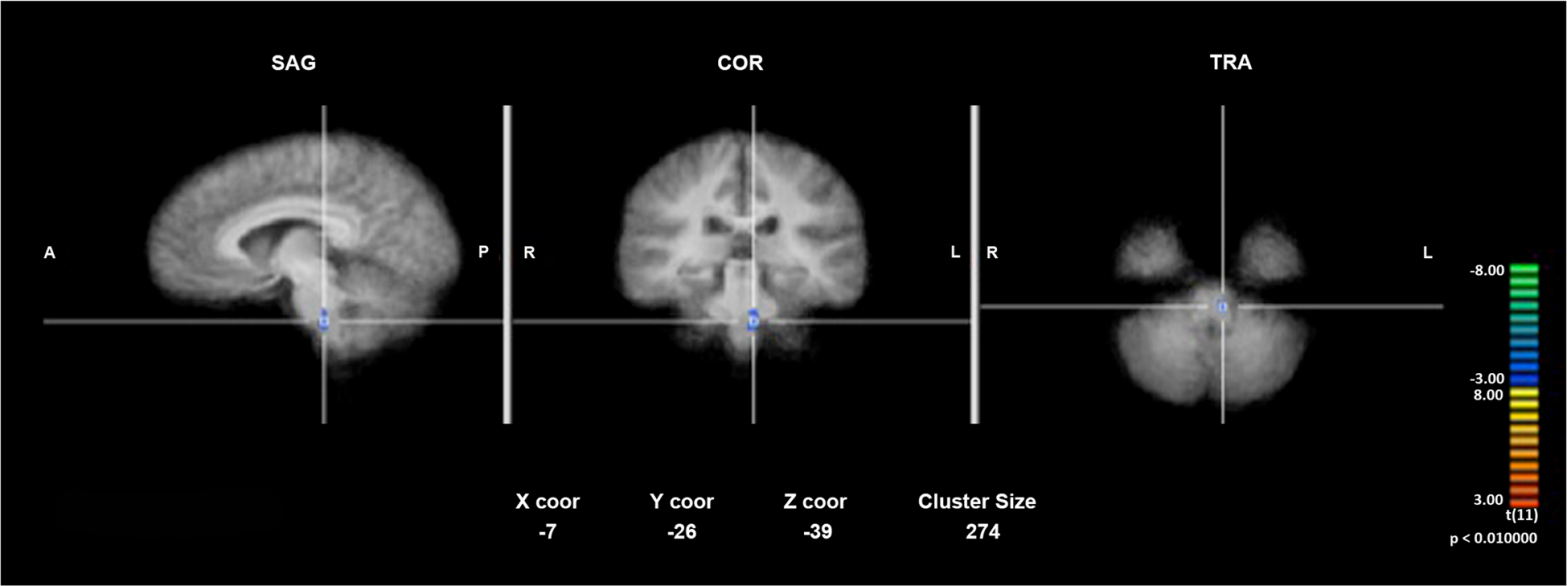
Deactivation seen in the medullary-pontine area (marked with a cross-pin on a normalized group averaged anatomical image) with the EA3>baseline within-group random effect analysis.

**Figure 3:**
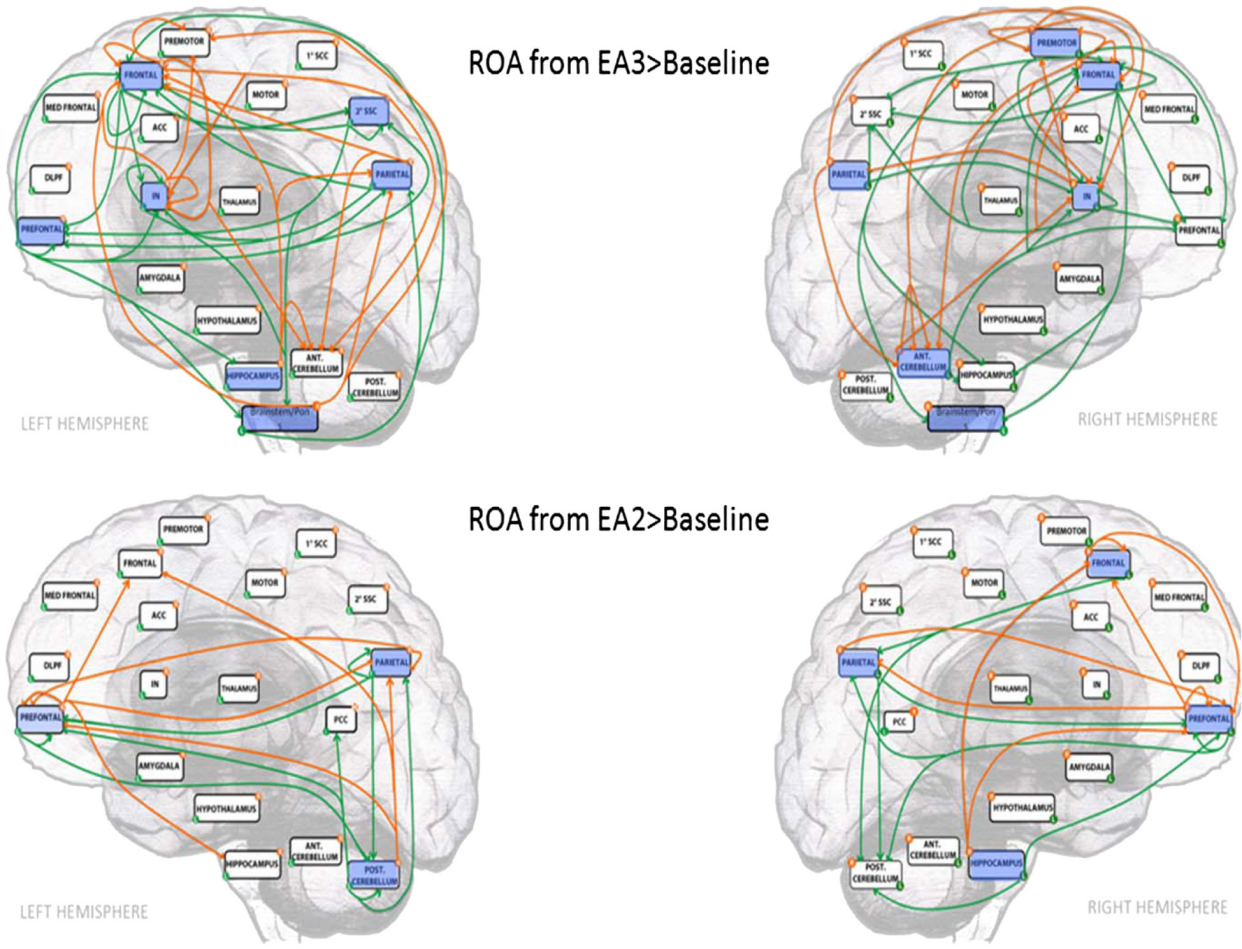
**Comparison of Granger Causality Analysis inference among regions of activities (ROA) extracted from EA3>Baseline and EA2>Baseline.** Blue box color indicates deactivation; Green arrow signifies inference originating from the left hemisphere, whereas orange arrow signifies inference originating from the right hemisphere; 1°SCC; Primary Somatosensory Cortex; 2°SCC: Secondary Somatosensory Cortex; ACC: Anterior Cingulate Cortex; PCC: Posterior Cingulate Cortex; DLPFC: Dorsolateral Prefrontal Cortex; IN: Insular Cortex; Med: Medial; Ant: Anterior; Post: Posterior.

In the correlated fMRI studies, both EA2>baseline (condition EA2 subtracted by baseline condition) and EA3>baseline (condition EA3 subtracted by baseline condition) resulted in a significant degree of deactivation in HP related regions including the inferior parietal lobe (IPL) and secondary somatosensory cortices (SSC2). The overall result as shown in Tables 1 and 2 suggested both paradigms were effective in modulating supraspinal pain processing. Furthermore, within-group random effect analysis indicated that EA3+HP>EA3 (condition EA3+HP subtracted by condition EA3) appeared to exert a significant degree of activity suppression in the affective supraspinal regions including the IPL, anterior cingulate cortex (ACC) and the insular cortex (IN). This level of suppression was not observed in the EA2+HP>EA2 (condition EA2 + HP subtracted by condition EA2) within-group analysis (see Figure 1), suggesting a mechanistic difference in the supraspinal pain modulating mechanisms between the two EA paradigms. Granger Causality Analysis (GCA) showed that EA3 induced a much higher degree of inference among supraspinal HP related somatosensory, affective and modulatory components than EA2. Specifically, the deactivation pattern at the medullary-pontine area in EA3 (Figure 2) contributed a direct inference on the deactivation pattern in SSC2 and IN (see Figure 3), suggesting a direct ascending pain modulatory effect induced by EA3.

**Table 1:** Regional of activities extracted from EA2>baseline (P<0.01, clutster >150 voxels); SSC2: secondary somatosensory cortex

**Hemisphere**	**Region of activity**	**T-value**	**Cluster size**	**Brodmann area**	**Peak coordinates**	**P-value**
**Left hemisphere**	***Sensory/Discriminatory***					
	**SSC2**	−12.529	299421	7	−31, -50, 45	<10^−6^
	***Affective & Emotional***					
	**Insular cortex**	−6.809	860	13	−40, 1, 9	0.000029
	**Hippocampus**	−5.4712	1670	N/A	−28, -11, -21	0.000194
	***Response & Neuromodulatory***					
	**Prefrontal cortex**	−8.904	13870	9	−25, 52, 30	0.000002
	**Prefrontal cortex**	−6.004	308	11	−31, 28, -24	0.000089
	**Prefrontal cortex**	−4.462	283	10	−4, 67, 18	0.00096
	**Frontal cortex**	−4.265	352	8	−19, 28, 48	0.001332
	**Brainstem/Pons**	−3.993	274	N/A	−7, -26, -39	0.002112
	**Frontal operculum**	−3.953	477	44	−49, 13, 12	0.002263
	**Prefrontal cortex**	−3.930	189	10	−7, 61, -3	0.002353
**Right hemisphere**	***Sensory/Discriminatory***					
	**Parietal**	−5.089	405	38	50, 25, -24	0.00035
	**Parietal**	−4.723	169	38	56, 10, -12	0.000627
	**Parietal**	−4.597	242	38	29, 22, -27	0.000769
	**Parietal**	−4.579	154	38	41, 16, -9	0.000792
	***Affective & Emotional***					
	**Insula cortex**	−4.170	529	13	35, 13, -3	0.001563
	***Response & Neuromodulatory***					
	**Frontal cortex**	−8.442	4409	8	26, 16, 36	0.000004
	**Cerebellum**	−5.831	589	Cerebellum	23, -29, -30	0.000114
	**Post. cerebellum**	−5.097	286	Claustrum	35, -14, 12	0.000346
	**Frontal operculum**	−4.680	1369	45	44, 22, 12	0.000671
	**Premotor cortex**	−4.033	201	6	8, 31, 54	0.001971

**Table 2:** Regional of activities extracted from EA3>baseline (P<0.01, clutster >150 voxels)

**Hemisphere**	**Region of activity**	**T-value**	**Cluster size**	**Brodmann area**	**Peak coordinates**	**P-value**
**Left hemisphere**	***Sensory/Discriminatory***					
	**Parietal**	−5.582	214	38	−55, -8, -24	0.000165
	**Parietal**	−5.362	400	38	−43, -2, -36	0.00023
	**Parietal**	−5.133	175	38	−43, -11, -36	0.000327
	**Parietal**	−4.627	181	38	−49, 7, -27	0.000731
	**Parietal**	−4.129	377	38	−49, 10, -6	0.001676
	**Affective & Emotional**					
	**PCC**	−11.772	216789	30	−10, -65, 9	<10^−6^
	***Response & Neuromodulatory***					
	**Cerebellum**	−5.531	335	N/A	−43, -77, -21	0.000178
	**Cerebellum**	−4.255	330	N/A	−7, -77, -15	0.001355
	**Prefrontal**	−4.209	310	9	−13, 58, 33	0.001462
**Right hemisphere**	***Sensory/Discriminatory***					
	**Parietal**	−5.687	203	38	53, 16, -15	0.000141
	***Affective & Emotional***					
	**Hippocampus**	−5.684	2942	N/A	29, -23, -15	0.000141
	***Response & Neuromodulatory***					
	**Frontal operculum**	−5.505	319	47	35, 28, -9	0.000185
	**Prefrontal cortex**	−5.164	164	9	11, 64, 30	0.000311
	**Prefrontal cortex**	−4.979	166	11	29, 22, -18	0.000416
	**Frontal operculum**	−4.8674	267	45	50, 25, 9	0.000497

Between-group random effect analysis indicated EA3 induced a significantly (P < 0.01, cluster size threshold > 150) higher degree of deactivation than EA2 in five HP related supraspinal areas including the right prefrontal cortex, rostral anterior cingulate (rACC), medial cingulate cortex, left inferior frontal lobe and posterior cerebellum. A two-factor (HP x EA) ANOVA in those regions indicated both rACC and posterior cerebellum had a significant (P ≤ 0.01) needle effect, and the right prefrontal lobe showed a significant (P < 0.01) HP effect. However, a significant interaction between the two factors was only found in the right prefrontal lobe.

## Discussion

Empirical acupuncture treatment often requires the use of multiple needles in various locations [10,11]. However, the exact functions and related neuronal mechanisms of different needling groups used in these paradigms are often not well defined. In addition, this requirement also creates a confounding factor in assessing the specific neuronal functions relating to a particular location/group of needle manipulation. The TMM is a well known and frequently used clinical acupuncture paradigm for treating acute pain [2]. The paradigm requires the stimulation of two groups of acupuncture points called the TPs and GP in order to provide analgesia for the extremities. A pervious study demonstrated a short duration of EA at the two lower extremities TPs (LR1 and SP1) could induce noticeable pain threshold elevation at the ipsilateral site of the stimulation in the correlated dermatome [12]. Another study established that extending the duration of stimulation increased the extent of the analgesic area [13]. In addition, a correlated fMRI study indicated that the observed analgesic effect on HP was closely related to a deactivational effect in HP related supraspinal areas [4]. In assessing the effect of needle combination, adding the GP stimulation to the TPS paradigm appeared to extend the analgesic duration and area [5]. The current study further compared the correlated supraspinal mechanisms of the two needle combinations within the TMM treatment paradigm.

In the area of thermal pain processing, it is well appreciated that at the spinal cord level, the ascending pain pathways mainly consist of the spinothalamic and the spinorecticular tracts [14]. The second order neurons of the spinothalamic tract decussate at the level of the spinal cord and travel up the length of the spinal cord and synapse with third order neurons at several nuclei of the thalamus (TH) including the medial dorsal, ventral posterior lateral, and ventral medial posterior nuclei. From there, signals go to the cingulate cortex, the primary somatosensory cortex, and IN respectively [15]. Unlike most ascending tracts, the spinoreticular tract consists of four levels of neurons. The tract begins with first-order neurons, which immediately synapse with second-order neurons in the posterior horn of the spinal column. These neurons decussate to the opposite side (anterolateral), and travel up the spinal column. It terminates in the brainstem at the medullary-pontine reticular formation. Information is sent from there to the intradmedian nucleus of the thalamic intralaminar nuclei. The thalamic intralaminar nuclei project diffusely to entire cerebral cortex where pain reaches conscious level and promotes behavioral arousal [15,16]. At the supraspinal level, recent studies with peripheral sensory testing and functional imaging techniques have provided insightful information regarding areas of the central nervous systems involved in encoding acute and chronic pain [17-21]. These supraspinal regions include the primary and secondary somatosensory cortices (SSC1 and SSC2), TH, IN, amygdala (AMG), and prefrontal cortices (PFCs). These supraspinal regions can further be functionally divided into the lateral and medial systems [22-26]. The lateral system, which is thought to be responsible for the initial noxious signal encoding, consists of the SSC1 and SSC2. The medial system, composed of the ACC and IN, is thought to underlie the affective component of the pain experience and direct the modulatory response. Both systems are mediated via the TH [27,28]. In addition other supraspinal regions such as the PFCs are known to play a crucial role in pain modulation [29-32]. In the area of acute HP perception, several supraspinal regions (TH, SSC1, SSC2, IPL, ACC, IN, PFCs) were consistently being implicated [7]. This level of understanding in the supraspinal mechanisms leading to the acute HP perception and modulation provides a framework for assessing the result of the current study.

In this study, both TMM treatment paradigms (EA2 and EA3) were effective in modulating HP. However, the treatment consisting of both TPs and GP stimulation (EA3) was more effective in modulating HP perception than the TPs stimulation alone (EA2) as reflected by the significant difference in the post-EA HP VAS scores between the two paradigms. This behavioral observation coincided with the findings of fMRI studies in which both paradigms resulted in a significant degree of deactivation in HP related supraspinal regions. However, EA3 induced a significantly higher degree of deactivation than EA2 specifically in five pain related supraspinal areas including the right prefrontal cortex, rostral anterior cingulate (rACC), medial cingulate cortex, left inferior frontal lobe and posterior cerebellum as shown in the between-group random effect analyses. Moreover, the additional GP (CV2) stimulation caused an appreciable decrease of activities at the medullary-pontine regions, suggesting a neuronal pain modulatory effect on the afferent/ascending signals in the spinoreticular pathways which were known to play an important role in the behavioral arousal of pain perception in the supraspinal area [16]. As shown in the GCA, the deactivation at the medullary-pontine region along the spinoreticular tract casted a direct inference in the diminished activity in SSC2 regions which in turn led to the deactivation in the IN known to be associated with the affective aspect of pain perception. This inhibitory inference initiating from the medullary-pontine region was not observed with TPs stimulation alone, suggesting the added GP stimulation exerted an inhibitory effect on the ascending spinoreticular pathway and augmented the analgesic effect of TPs stimulation.

## Conclusion

EA of the TPs in the TMM treatment paradigm diminished activities in supraspinal regions normally associated with HP activation. The addition of GP stimulation to the TPs further enhanced the analgesic effect. This augmented analgesic effect appeared to be mediated via the ascending spinoreticular pain pathway causing diminished activities in the SSC2 and IN when both TPs and GP were stimulated. Therefore, different needling locations as mandated in the empirical TMM treatment paradigm appear to have a different modulatory effect on the pain pathways.

## Methods

With the Institution Human Subject Protection Committee approval, healthy volunteers were enrolled for the studies based on the following inclusion and exclusion criteria:

Inclusion criteria: Age 18 to 80; Male and female; No analgesics for the past 2 weeks; Absence of neuropathic pain states;

Exclusion criteria: History of psychological illness; History of claustrophobia; Lack of ability to understand the experimental protocol and to adequately communicate in English; Pregnancy; Pending litigation; History of head trauma, history of trauma or surgery to lower extremities or low back; History of any metallic implant in the body as listed in the Institute fMRI Center screening list.

### Pre-scanning neurosensory threshold assessment

To be consistent in the study, the location of the thermal thresholds measurement and stimulation was marked at the medial aspect of the left calf between the 6th and 7th marking of an elastic band which consisted of a total of 13 increments, extending from the medial malleolus to the medial tibial plateau. To ensure that the baseline thermal sensory thresholds of the study cohort were within normal range, non-noxious and noxious thermal thresholds including cold and warm, cold and hot pain were measured by using a Thermal Sensory Analyzer (Medoc Advanced Medical Systems, Minneapolis). This device consisted of a thermode measuring 46 × 29 mm. The temperature of the thermode could either rise or fall (at a rate of 1.2 degrees Celsius/sec for cold and warm sensations, and 3 degrees Celsius/sec for cold and hot pain), depending on the sensations that were being tested. The subject signaled the onset of feeling the tested sensation by pressing a switch, which in turn reversed the temperature change and returned the temperature of the thermode to the 32 degree Celsius baseline. The computer then recorded the temperature of the thermode when the switch was pressed. The average value of testing result was automatically calculated by the computer and displayed on the screen. For the pre-scan HP VAS score assessment, a 15-second subject threshold specific HP stimulus was given the mid calf, the intensity of the pain was recorded on a VAS. This method of peripheral sensory testing has been well established in literature and has been used extensively in pain-related studies [3,8,13,33,34].

### fMRI scanning

Subjects were placed comfortably in a supine position in a scanner with their eyes covered by an eye shield. A Facial-cervical Collar Restraint (FCCR) Device was applied to minimize head movement [35]. The following fMRI scans were performed in a random order:

1) 60 seconds of baseline thermal stimulus at 32°C, followed by 15 seconds of subject specific HP stimulation at the left medial calf.

2) After the initial 15-second EA (EA2, EA3), HP was delivered at the medial calf for 15 seconds (EA2+HP, EA3+HP). The stimulus was separated by 60 seconds of baseline thermal stimulus at 32°C.

**Figure 4:**
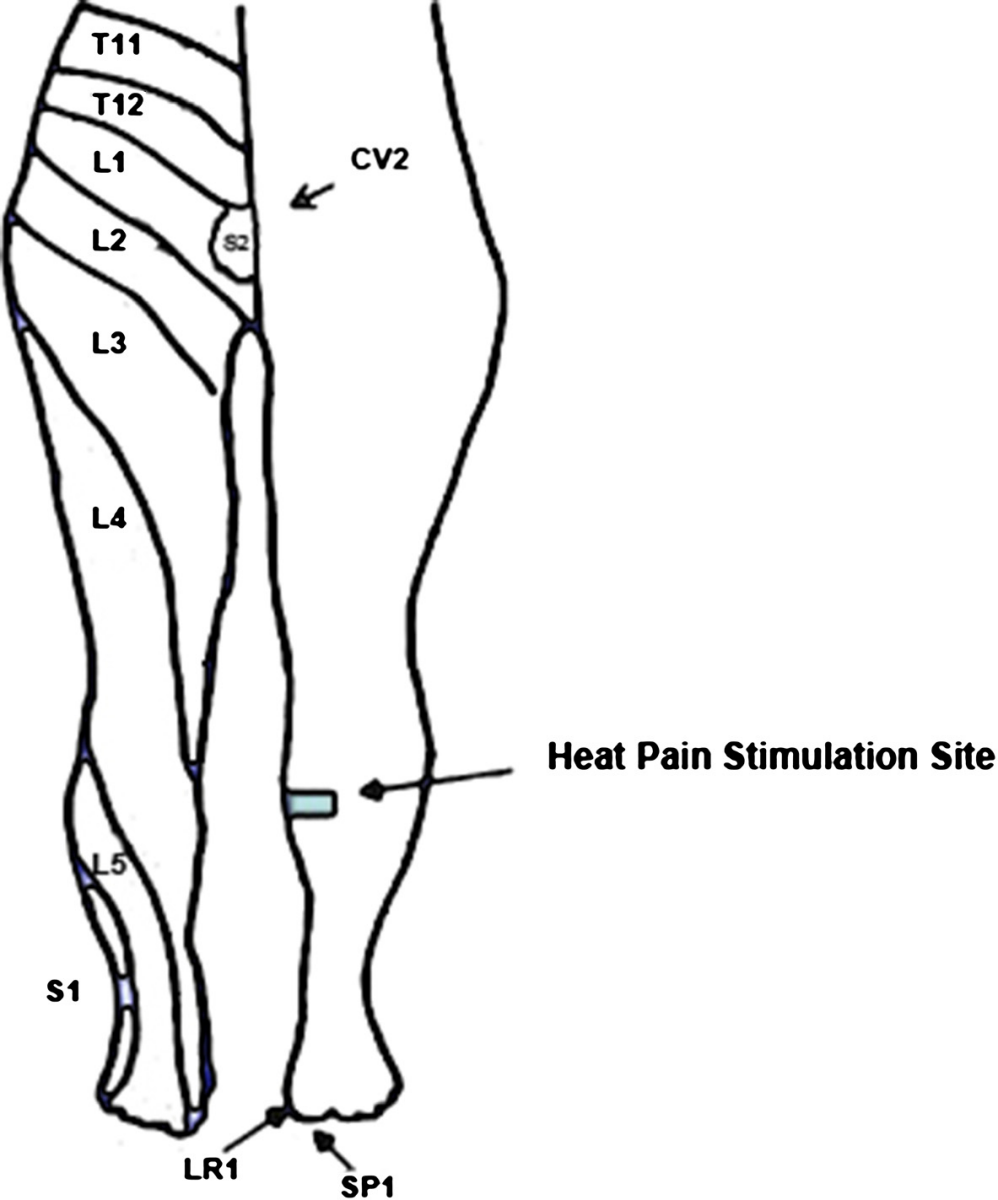
**Location of Ting points (LR1 and SP1) and Gathering Point (CV2) in the Tendinomuscular Meridian System.** LR1: Liver 1 (Big Mound); SP1: Spleen 1 (Yinbai); CV2: Conception Vessel 2 (Qugu Ren-2).

All stimulus and/or EA sequence was repeated 4 times to complete the sequence. The 2-needle EA(EA2) of the TMM (see Figure 4) consisted of 5 Hz stimulation optimally adjusted to the subjects’ de qi sensation at SP 1 (Yinbai), LR 1 (Big Mound), whereas the 3-needle EA (EA3) consisted of an additional needle at CV 2 (Qugu Ren-2). Acupuncture needles were placed at following locations:

1) SP1 (Yinbai) --on the dorsal aspect of the big toe, at the junction of lines drawn along the medial border and the base of the nail, approximately 0.1 cun from the corner of the nail [36].

2) LR1 (Big Mound)--on the dorsal aspect of the big toe, at the junction of lines drawn between the lateral border and the base of the nail, approximately 0.1 cun from the corner of the nail [36].

3) CV2 (Qugu Ren-2)—at the superior border of the pubic symphysis along the midline of the lower abdomen, 5 cuns below the umbilicus [36].

One-inch-long, 36G fMRI-compatible gold plated sterile needles were used. The location and method of needle placement used in the study were described in the previously published studies [3,13]. Both the needles and grounding electrodes were linked to a 6-volt ES-160 (Electro-Therapeutic Devices Inc., Markham, Ontario, Canada) clinical acupuncture stimulation device, which consisted of a digital display of the stimulation paradigm. Electrical stimulation was provided at a constant frequency of 5 Hz with a pulse width of 300 microseconds. The needle placement and stimulation intensity was adjusted based on the method used in previous published study [4]. At the end of the scanning session, the subjects were asked to rate the overall heat pain score on a VAS score. All needle placements were conducted by an experienced medical acupuncturist.

In between each scanning paradigm, a minimal of 15 minutes of washout period was provided to ensure either the HP sensation or the EA related deqi sensation has completely subsided.

FMRI Images were obtained via a 3 T GE scanner with T2*- weighted EPI-sequence (TE = 30 ms, TR = 2.0 s, α=90°, TH = 4 mm, 32 slices, FOV = 220×220 mm^2^, MA = 64×64). Two T1-weighted images were acquired: one for spatially normalizing the functional images and the other one for anatomical details.

### Behavioral data analysis

A paired sample *t*-test was used to compare the VAS scores of hot pain and *de qi* sensations.

### fMRI data analysis

Each individual subject’s functional and anatomical data sets were processed, aligned and prepared in Brain Voyager for within- and between group random effects analyses based on steps described by Goebel et al. [37].

### Preprocessing of functional data

Raw functional data (dicom format) was loaded and converted into Brain Voyager’s internal “FMR” data format. Standard sequence of preprocessing steps including slice scan time correction, head motion correction, drift removal and spatial smoothing with Gaussian filter (FWHM = 5 mm) were conducted for each paradigm data set of each subject.

### Preprocessing of anatomical data

The anatomical data (dicom format) of each subject was loaded and converted into Brain Voyager’s internal “VMR” data format. Intensity inhomogeneities correction as applied and the data were then resampled to 1-mm resolution, and transformed into AC-PC and Talairach standard space. The three spatial transformations were combined and applied backward in one step to avoid quality loss due to successive data sampling. The two affine transformations, iso-voxel scaling and AC-PC transformation, were concatenated to form a single 4X4 transformation matrix. For each voxel coordinates in the target (Talairach) space a piece affine “Un-Talairah” step was performed, followed by application of the inverted spatial transformation matrix. The computed coordinates were used to sample the data points in the original 3-D space using sinc interpolation.

### Brain segmentation

For 3-D visualization, the brain was segmented from surrounding head tissue using an automatic “brain peeling” tool. The tool analyzes the local intensity histogram in small volumes (20×20×20 voxels) to define thresholds for an adaptive region-growing technique. This step resulted in the automatic labeling of voxels containing the white and gray matter of the brain, but also other high-intensity head tissue. The next step consisted of a sequence of morphological erosions to remove tissue at the border of the segmented data. By “shrinking” the segmented data, this step separated subparts, which were connected by relatively thin “bridges” with each other. By determining the largest connected component after the erosion step, the brain was separated from other head tissue. Finally, the sequence of erosions was reversed but restricted to voxels in the neighborhood of the largest connected component.

### Cortex segmentation

In order to perform a cortex-based data analysis, the gray/white matter boundary was segmented using largely automatic segmentation routines [38]. Following the correction of inhomogeneities of signal intensity across space as described above, the white/gray matter border was segmented with a region-growing method using an analysis of intensity histograms. Morphological operations were used to smooth the borders of the segmented data and to separate the left from the right hemisphere. Each segmented hemisphere was finally submitted to a “bridge removal” algorithm, which ensures the creation of topologically correct mesh representations [38]. The borders of the two resulting segmented subvolumes were tessellated to produce a surface reconstruction of the left and right hemisphere. For better visualization of the areas of activities including those in the sulcus, the resulting meshes were transformed into inflated cortical representations by performing repeated small morphing steps until the central sulcus are visible. The inflated cortical meshes were used as the reference meshes for functional data (maps and time courses) projection. For subsequent cortex-based analysis, the inflated cortical meshes were used to sample the functional data at each vertex (node), resulting in a mesh time course (“MTC”) dataset for each run of each subject.

### Normalization of functional data

To transform the functional data into Talairach space, the functional time series data of each subject was first coregistered with the subject’s 3-D anatomical dataset, followed by the application of the same transformation steps as performed for the 3-D anatomical dataset (see above). This step resulted in normalized 4-D volume time course (“VTC”) data. In order to avoid quality loss due to successive data sampling, normalization was performed in a single step combining a functional-anatomical affine transformation matrix, a rigid-body AC-PC transformation matrix, and a piecewise affine Talairach grid scaling step. As described for the anatomical normalization procedure, these steps were performed backward, starting with a voxel in Talairach space and sampling the corresponding data in the original functional space. In the context of the functional-anatomical alignment, some manual adjustment was necessary to reduce as much as possible the geometrical distortions of the echo-planar images, which exhibited linear scaling in the phase-encoding direction. The necessary scaling adjustment was done interactively using appropriate transformation and visualization tools of Brain Voyager QX.

### GLM analysis

For each run of each subject’s block, a protocol file (PRT) was derived representing the onset and duration of the events for the different stimulation conditions. In order to account for hemodynamic delay and dispersion, each of the predictors was derived by convolution of an appropriate box-car waveform with a double-gamma hemodynamic response function [39] to extract brain regions with both positively and negatively correlated blood oxygen level dependent (BOLD) responses. Within group random effect analysis was conducted for each paradigm and areas of activation (positively correlated BOLD) and deactivation (negatively correlated BOLD) were recorded. Between-group random effect analyses were also performed between EA2 and EA3 paradigms and a second level 2-factor ANOVA (EA and GP stimulation) was also performed to assess effect of treatment and GP stimulation interaction at regions of interest related to HP stimulation.

Within-group random effect analysis was conducted to assess the supraspinal effect of the two EA paradigms (EA2>Baseline, EA3>Baseline), and the effect of EA on HP (EA2+HP>EA2, EA3+HP>EA3).

Granger Causality analysis (GCA) was conducted to explore the causal interaction (inference) among regions related to pain perception for EA2 and EA3. First, the affected regions in the form of either activation or deactivation from the HP paradigm were used to create a cluster-based (anatomically based) template for the intended GCA. Each of the regions was used to estimate effective connectivity among clusters in each paradigm with the BV GCA plug-in. Using the average time course in one of the regions as a reference and the other regions as potential target regions of inference, computations were made to discern the correlation of the voxels in these regions from the rest of the brain. The result of the analysis was displayed as either positive values signifying significant influence directing from the reference cluster to the targeted regions or negative values representing the reverse direction. In addition, clusters information including coordinates, sizes and Brodmann areas were converted by the Talairach Client into a text format after verifying the data with Brain Tutor [40,41]. The resulting text was imported to a spreadsheet and the network of inference was mapped onto a spatial representation of the brain network involved in acute thermal pain processing for each paradigm. Between-group (EA3>EA2) analysis was conducted to assess ROAs that were significantly different between the two paradigms. Those ROAs were then subjected to 2-factor (HP, Needle) ANOVA for between-group (EA2+HP>EA2, EA3+HP>EA3) comparison.

## Abbreviations

ACC: Anterior cingulate cortex; ANOVA: Analysis of variance; BA: Brodmann area; BOLD: Blood oxygen level dependent; BV: Brain voyager; CV2: Conception vessel 2 (Qugu Ren-2); DLPFC: Dorsolateral prefrontal cortex; EA: Electroacupuncture; EA2: Electroacupuncture with Ting Points; EA3: Electroacupuncture with Ting points and Gathering point; EA2>Baseline: Condition EA2 subtracted by baseline condition; EA3>Baseline: Condition EA3 subtracted by baseline condition; EA2+HP>EA2: Condition EA2 subtracted by condition EA2; EA3+HP>EA3: Condition EA3 subtracted by condition EA3; FDR: False discovery rate; FMRI: Functional magnetic resonance imaging; fMRI: Functional magnetic resonance imaging; FO: Frontal operculum; GCA: Granger Causality Analysis; GLM: General linear model; GP: Gathering point; HP: Hot pain; IN: Insular cortex; IPL: Inferior parietal lobe; LR1: Liver 1 (Big Mound); MPFC: Medial prefrontal cortex; MTC: Mesh time course; PFCs: Prefrontal cortices; POI: Patch of interest; SP1: Spleen 1 (Yinbai); SSC1: Primary somatosensory cortex; SSC2: Secondary somatosensory cortex; TH: Thalamus; TPs: Ting Points; VAS: Visual analogue scale; VOI: Volume of interest; VTC: Volume time course.

## Competing interests

The authors declare that they have no competing interests.

## Authors’ contributions

AL carried out the experiment, supervised the data analysis and prepared the manuscript. ZY carried the data analysis, graphic and manuscript preparation. SS conducted the data analysis. All authors read and approved the final manuscript.
